# Development of a co‐culture of *Ureibacillus thermosphaericus* and *Cupriavidus taiwanensis* for inhibitors removal from hemicellulose prehydrolysate

**DOI:** 10.1002/btpr.70107

**Published:** 2026-01-19

**Authors:** Mariem Theiri, Mariya Marinova, Hassan Chadjaa, Mario Jolicoeur

**Affiliations:** ^1^ Research Laboratory in Applied Metabolic Engineering, Department of Chemical Engineering Polytechnique Montréal, J.‐A.‐Bombardier Pavilion Montréal Quebec Canada; ^2^ Industrial Bioprocesses Centre National en Électrochimie et en Technologies Environnementales Shawinigan Quebec Canada; ^3^ Department of Chemistry and Chemical Engineering Royal Military College of Canada Kingston Ontario Canada

**Keywords:** co‐culture, detoxification, phenolic compounds, pre‐hydrolysate, synthetic media

## Abstract

For biofuels production, hemicellulose pre‐hydrolysate is considered an attractive feedstock rich in fermentable sugars. The lignocellulosic biomass comprises, along with sugars, several inhibitors that can hamper its efficient conversion. In this work, mixed cultures of *Ureibacillus thermosphaericus* and *Cupriavidus taiwanensis* were used for the first time to detoxify the pre‐hydrolysate. The nutrient source was first optimized in synthetic media with mono‐cultures to detoxify phenolic compounds, and a medium containing inorganic salts was selected. Afterwards, the efficiency of phenolic degradation was compared in a single‐compound solution and in a mixture. The simultaneous co‐culture showed the highest degradation efficiency (90% at 2.8 g/L of phenolic compounds). Finally, the detoxification of a raw pre‐hydrolysate was conducted, and a maximum degradation of 14% of the phenolics was obtained using sequential inoculation of *Ureibacillus thermosphaericus* followed by *Cupriavidus taiwanensis* addition.

## Graphical abstract

For biofuels production, hemicellulose pre‐hydrolysate is considered as an attractive feedstock rich in fermentable sugars. The pre‐hydrolysate derived from lignocellulosic biomass comprises, along with sugars, several inhibitors which can hamper its efficient conversion. In this work, mixed cultures of *Ureibacillus thermosphaericus* and *Cupriavidus taiwanensis* were used for the first time to detoxify the pre‐hydrolysate. The nutrient source was first optimized in synthetic media with mono‐cultures to remove phenolic compounds, and a medium containing inorganic salts was selected. Afterwards, the efficiency of phenolic degradation was compared in a single‐compound solution and in a mixture. The simultaneous co‐culture showed the highest degradation efficiency (90% at 2.8 g/L of phenolic compounds in a mixture). Finally, the detoxification of a raw pre‐hydrolysate was conducted, and a maximum degradation of 14% of the phenolics was obtained using sequential inoculation of *Ureibacillus thermosphaericus* followed by *Cupriavidus taiwanensis* addition.

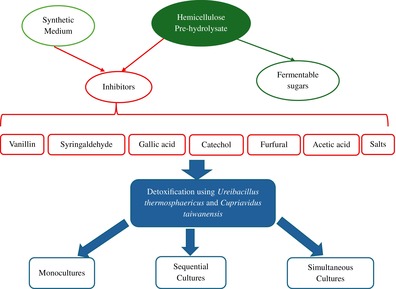

## 1 INTRODUCTION

Lignocellulosic biomass is characterized by its annual availability, sustainability, renewability, low cost, and non‐food criterion.^1^, ^2^, ^3^, ^4^ This biomass is considered as an attractive feedstock for biofuels production. However, the recalcitrant nature of lignocellulose, due to its complex structure, hampers the access of enzymes and chemicals to the carbohydrates they need to liberate fermentable reducing sugars.^4^, ^5^ To break the complex structure of the biomass and thus maximize the yield of fermentable sugars and the production of bioenergy and bio‐based products, pre‐treatment and hydrolysis steps are required.^4^ Various biomass pretreatment processes, among others acid hydrolysis, hot water extraction and steam explosion, have been developed.^4^, ^6^, ^7^ Depending on the process conditions, several inhibitors covering a broad concentration range are also released along with fermentable sugars.^4^, ^5^, ^8^

During acid hydrolysis, different phenolic compounds are generated through the cleavage of β‐O‐4 ether and other acid–labile linkages present in lignin. Gallic acid is generated from hydrolysable tannins in hardwood lignocellulose biomass.^9^, ^10^ but can also be obtained from enzymatic hydrolysis through hydroxylation of protocatechuate derivative. Protechatechuate is obtained from o‐demethylation, and oxidation of aromatics from lignin depolymerization.^11^

Catechol is usually generated from thermal pretreatment processes, through the ether cleavage of phenolic methoxyl groups.^12^

The toxic compounds, released during hydrolysis together with the sugars, are also part of the structure of the lignocellulosic biomass.^13^ Phenolic compounds are considered major inhibitors, and examples of these products are vanillin, catechol, gallic acid, syringaldehyde, *p*‐coumaric acid, and ferulic acid, which decrease the performance of enzymatic hydrolysis.^13^, ^14^, ^15^ Gallic acid is a trihydroxybenzoic acid composed of three hydroxyl groups and one carboxylic acid group. Gallic acid can naturally occur in several plant families (e.g., *Myrtaceae*, *Anacardiaceae*) and fruits (e.g., grapes, mangoes, tea). It is also present in non‐sugar galloyl esters of gallic acid. It is synthesized in plants through the shikimate pathway. Gallic acid can also originate from fungi of the *Termitomyces* genus and can occur in nature as a constituent of hydrolysable tannins.^16^

Catechol is a benzenediol comprising two hydroxyl groups.^17^ This inhibitor is a central intermediate in the degradation pathways of lignin‐derived aromatic compounds (e.g., ferulic acid, *p*‐coumaric acid, protocatechuate).^18^ Catechol also occurs naturally in plants from hydroquinone oxidation.^19^

As for vanillin, its corresponding functional groups are hydroxyl, aldehyde, and ether groups. For syringaldehyde, it is a hydroxybenzaldehyde that includes one hydroxyl, two ether, and one aldehyde groups.

These structurally different phenolic compounds are also different in their toxicity. Syringaldehyde and vanillin, generated during treatment of lignocellulose,^20^, ^21^, ^22^ have been shown to be potential inhibitors of *C. acetobutylicum* on both bacterial growth and butanol yield.^20^, ^23^ Cho et al. demonstrated that these compounds are less toxic to the growth of *C. beiijerinckii*, but they completely inhibited butanol production.^24^ On their tour, Ibraheem and Ibraheem claimed that aldehyde and/or carboxylic acid groups are more toxic than alcohol groups.^25^ Vanillin and syringaldehyde contain an aldehyde group; gallic acid has a carboxylic acid group and catechol is an alcohol group phenolic compound.^26^ In parallel, toxicity is related to the molecular weight of the phenolic compound: The lower the molecular weight, the higher its toxicity.^23^, ^27^ Then, molecular weights of phenolic compounds are in decreasing order: syringaldehyde, gallic acid, vanillin, and catechol. Therefore, the degree of toxicity of phenolic compounds depends on several factors, among others the microorganism, the biomass containing the phenolics, the concentration in the medium, and so forth.

Balasundaram et al. claimed that low molecular weight of phenolic compounds facilitates the penetration through cell membranes, and they harm their internal structures. These inhibitors are of higher toxicity for anaerobic cultures than the other toxic compounds.^7^

The phenolics are also the major inhibitors of hydrolysis^28^ reduced by half the activity of ß‐glucosidase from *Trichoderma reesi* as reported in the work of Kim et al.^14^ Besides their impact on cellulolytic enzymes,^21^, ^29^ phenolics can decrease cell growth and solvent production yields.^30^, ^31^ The cell metabolism was totally blocked, and no production of Acetone‐Butanol‐Ethanol (ABE) was observed in the presence of 1 g/L of syringaldehyde, *p*‐coumaric acid and ferulic acid, as claimed by Yao et al.^32^ In the work of Ezeji et al.,^29^ a concentration of 0.5 g/L of ferulic acid increased the lag phase and decreased butanol production with *Clostridium beijerinckii* NCIMB 8052 up to 90% and the cell growth by 80%. Phenolic compounds interfere with the cell membrane of the solventogenic cells and increase its fluidity leading to the leak of cellular contents.^27^, ^28^, ^30^ Vanillin, ferulic acid and syringaldehyde are responsible for membrane integrity loss of *S. cerevisae*. Levulinic acid can alter membrane integrity of *S. cerevisae*, then lead to inhibition of fermentation and respiration through pH decrease and ATP depletion.^33^ Kim et al. demonstrate that vanillin can inhibit mitochondrial functions for fungi and cause oxidative stress leading to reduced growth and proteolysis.^34^

In addition to phenolics, the inhibition of cell growth can be caused by Furfural and 5‐hydroxymethyl furfural (5‐HMF) (totally inhibited in the presence of 4 g/L of furfural)^35^ resulting from the degradation of pentose and hexose sugars, respectively during pretreatment and hydrolysis.^29^ ABE production by *Clostridium acetobutylicum* ATCC 824 decreased down to 10% at 3 g/L of furan aldehydes.^27^ Furthermore, furfural and 5‐HMF contribute to redox imbalance and inhibition of glycolysis, protein and RNA synthesis, and ethanol fermentation. Furans are also responsible for DNA damage, ROS accumulation and thus cellular component damage.^9^

However, several researchers have demonstrated that the presence of low concentrations of furan aldehydes (furfural and HMF) (i.e., up to 2 g/L) has a stimulatory effect on the overall cell growth and butanol production by *Clostridium* species. During ABE fermentation, furfural and HMF aldehydes are converted to their corresponding alcohols which are less inhibitory than the aldehydes.^31^, ^32^, ^35^ The biotransformation of furan aldehydes regenerates NAD^+^, enhancing the glycolysis yield which improves cell growth.^32^

Acetic acid is another inhibitor generated from the break‐down of acetyl groups in hemicelluloses.^36^, ^37^ A concentration of 60 mM of acetic acid induced at its undissociated form an “acid crash” during ABE fermentation with *Clostridium beijerinckii* B592, which could contribute to cell death.^30^, ^36^ Camara and al.^35^ claimed that undissociated weak acids penetrate through the cell membrane and dissociate inside the cell, contributing to acidic conditions which require an increase in ATP consumption to maintain intracellular pH.

Treatment of lignocellulosic material with alkali and acid solutions during pretreatment, hydrolysis or neutralization steps could also release inorganic salts, for example sodium sulfate and sodium chloride,^36^, ^38^ potential inhibitors of the ABE fermentation. Concentrations of sodium chloride higher than 10 g/L inhibited *Clostridium beijerinckii* BA101.^39^

The development of a detoxification procedure is critical to counteract the inhibition and maximize the conversion of hemicelluloses into value added products. Several detoxification methods were applied to alleviate the inhibitory effects of toxic compounds. Among others, resin treatments, activated charcoal,^40^ tannin‐based biopolymers,^41^ chitosan‐chitin nanofiber beds,^42^ ammonia, electrodialysis, organosolv., dilution and filtration or centrifugation for solid/sediment removal.^43^ Metabolic engineering and mutations could equally improve strain tolerance to inhibitors, and then increase ABE yields.^44^ Sheng et al. added amino acids and proteins to reduce inhibitors generation during Aspen pretreatment. The authors demonstrated the high efficiency of detoxification by addition of histidine. Supplementation of the biomass with proteins equally improved toxic compounds removal by their reaction with aldehydes and ketones.^45^ However, these methods usually employ harsh operating conditions (high concentration of chemicals, high temperature, etc.) in addition to fermentable sugars losses exceeding 25% with anion‐exchange resin at pH 10^46^ and 30% with activated charcoal.^47^, ^48^

The side effects that negatively affect the economic viability of the process may be alleviated by means of biological treatment using ligninolytic enzymes extracted from microorganisms or applying these strains directly. Although enzymatic detoxification is very effective, leading to the degradation of 75% of the phenolic compounds in steam‐exploded wheat straw, this treatment, besides its high cost, targets only phenolic compounds^28^ without affecting the other inhibitors. An alternative to chemical, physical and enzymatic methods of detoxification is the microbial treatment employing bacteria or fungi.

Although these processes are effective, they considerably increase the overall complexity and cost of hydrolysate detoxification. In contrast, our approach relies on the naturally occurring oxidative activity of microorganisms to remove inhibitory compounds directly from the hydrolysate, thereby enhancing the economic feasibility and environmental sustainability of the process. Therefore, while the underlying oxidative reactions are well known, their direct microbial application for detoxifying hemicellulose hydrolysates represents a novelty and practical contribution to the development of more sustainable, cost‐effective, and integrated biorefinery processes.

This technique targets various inhibitors (phenolic compounds, furfural, 5‐HMF, acetic acid, etc.) and has a low cost and a positive environmental impact. Fungi cells express membrane transporters to eliminate the toxic compounds. Klosowski et al. demonstrate the ability of yeast cells and bacteria to reduce furfural and 5‐HMF to their corresponding alcohols, furfuryl alcohol and 2,5‐bishydroxymethylfuran respectively. Furthermore, overexpression of genes responsible for particular cellular responses could increase the tolerance of yeast cells to toxic compounds.^33^

However, most of the microorganisms are characterized by low detoxification yields, long residence time, and consumption of fermentable sugars. For instance, *Trichoderma reesei* consumed 35% of the fermentable sugars in a dilute acid hydrolysate of spruce, while detoxifying a maximum of 6% of the phenolic compounds for 6 days.^46^ Lignin loss in corn stover as high as 51.4% was reached when pretreated with *Phanerochaete chrysosporium* NRRL‐6370 for 1 month accompanied by hemicelluloses loss of 57%.^49^ *Cupriavidus taiwanensis* (*C. taiwanensis*) and *Ureibacillus thermosphaericus* (*U. thermosphaericus*) were proven to be among the most effective detoxification strains due to their high performance in short duration (24 h), without consumption of fermentable sugars.^50^ However, researchers demonstrated the low tolerance of *C. taiwanensis* to high concentrations of inhibitors. Many attempts, such as the use of fed‐batch cultures or supplementation of inducers of phenols degradation, to enhance the performance of the bacteria did not achieve a significant improvement of the detoxification of highly inhibitory biomass.^51^, ^52^, ^53^, ^54^ To enhance tolerance of strains, increase the yields of detoxification of a wider range of inhibitors and shorten the duration of treatment, the development of a co‐culture composed of both strains (*C. taiwanensis* DSM 17343 and *U. thermosphaericus* NCIMB 13819) was investigated for the first time in this work.

Recent studies further support the relevance of microbial detoxification of lignocellulosic hydrolysates through co‐culture or engineered strains. For instance, Ujor and Okonkwo review the biochemical mechanisms of microbial detoxification of furanic and phenolic inhibitors.^55^ Jilani and Olson^56^ and Shabbir et al.^57^ describe metabolic tolerance strategies to furfural and acetic acid in microorganisms, which align with our co‐culture approach. Furthermore, Xia et al.^58^ demonstrated the removal of phenolic inhibitors from corn‐straw hydrolysate to enhance bioproduct formation, and Li et al.^59^ explored microbial interactions in a detoxification‐fermentation system. Previous studies demonstrated that a co‐culture may affect the long duration of the biodetoxification process.^60^, ^61^

In this work, synthetic media containing the inhibitors present in conventional pre‐hydrolysates (steam explosion, acid, alkaline and hydrothermal methods, etc.) and a hemicellulosic pre‐hydrolysate generated in a Kraft dissolving pulp process were detoxified using an original co‐culture strategy. The consortium proved higher efficiency in the removal of a broader range of inhibitors than mono‐cultures. In a recent study performed by the authors (under submission), the optimal temperature favoring similar kinetics of mono‐ and co‐cultures was determined and a temperature of 41°C was fixed. In that study, the initial ratio of *C. taiwanensis* and *U. thermosphaericus* was evaluated to obtain an equal number of strains in the detoxification media. The Q‐PCR approach was used and a starting volumetric ratio of 1‐unit *Cupriavidus taiwanensis* per 1.5‐units *Ureibacillus thermosphaericus* was fixed.

The main purpose of this study was to evaluate the biodetoxification of lignocellulosic biomass using *Ureibacillus thermosphaericus* and *Cupriavidus taiwanensis*. Specifically, the objective of this work was to investigate the effect of the synthetic medium composition, representative for hemicellulose hydrolysate, on the efficiency of phenolic compound removal with pure and mixed cultures of *U. thermosphaericus* and *C. taiwanensis*.

*Ureibacillus thermosphaericus* is a thermophilic Gram‐positive bacterium that forms entire, circular and flat colonies^62^ and is well known for its high‐level production of catalase with very high enzymatic activity.^63^

*Cupriavidus taiwanensis* is a Gram‐negative β‐rhizobium, also identified as *Ralstonia taiwanensis* and *Burkholderia mimosarum* and is known for its ability to establish nitrogen‐fixing symbioses and for its potential in bioremediation and biodetoxification through tolerance to, and degradation of, various environmental contaminants. These features make *C. taiwanensis* a promising bioremediation and biodetoxification candidate.^64^

## 2 MATERIALS AND METHODS

### 2.1 Microorganisms, media and culture conditions

*U. thermosphaericus* NCIMB 13819 was obtained from NCIMB Ltd. (YA, Scotland) and was activated in 3% *m/v* sterilized Tryptic Soy Broth (TSB) (Quelab Laboratories inc.; QB‐39‐5626) at 55°C with agitation at 180 rpm until two deceleration points of Optical Density (OD_600_). Glycerol 30% *v/v* was then added to prepare a stock culture frozen at −80°C. For inoculum preparation, TSB (in g/L: casein peptone 17, soy peptone 3, dipotassium phosphate 2.5, dextrose 2.5 and sodium chloride 5) was autoclaved at 121°C for 15 min, then inoculated with 0.1% *v/v* of the stock culture. The culture was let to grow for 10 h at 50°C with agitation at 180 rpm in an orbital shaker (New Brunswick Scientific Innova 44, USA). The duration of the inoculum was determined from the growth curve of *U. thermosphaericus* incubated during 48 h on TSB. This time corresponds to the half of the maximum OD_600_ obtained (data not shown). Two mL of the inoculum were inoculated to the media where detoxification experiments were performed. The dose of inoculum corresponded to an initial concentration of 0.2 × 10^8^ CFU/mL.

The 48 h period corresponds to the growth‐curve analysis of *U. thermosphaericus* NCIMB 13819. Based on this growth curve, the strain reached its mid‐exponential phase at a time that corresponds to half of the maximum OD_600_ obtained (data not shown), and this time point was therefore selected for harvesting the inoculum, as microorganisms are most metabolically active and suitable for detoxification at this stage.

*C. taiwanensis* DSM 17343 was purchased from DSMZ (Braunschweig, Germany). This strain was revived in R2A medium, consisting of (in g/L): yeast extract 0.5 (BD, REF: 212750), proteose peptone 0.5 (Nutri‐Bact; QB‐39‐1910), casamino acids 0.5 (organotechnie), glucose 0.5 (Thermo Fisher Scientific D1610), soluble starch 0.5 (Fisher Chemical; S516‐500), Na‐pyruvate 0.3 (Fisher Bioreagents; 253 S‐15629U), K_2_HPO_4_ 0.3 (Fisher Bioreagents; catalog number 19 L‐15781U) and MgSO_4_.7H_2_O 0.05 (Acro Organics; catalog number 423905000). The strain was then incubated at 28°C and 180 rpm in an orbital shaker (Barnstead/Lab‐Line, MaxQ 4000 Orbital Shakers, USA) until two deceleration points of Optical Density (OD_600_). The stock culture was prepared from the R2A culture similarly to *U. thermosphaericus* as described earlier. For inoculum preparation, TSB was used and the inoculum was allowed to grow for 10 h at 37°C with agitation at 180 rpm. To inoculate the detoxification media, 1.33 mL of the inoculum was used. The dose of inoculum corresponded to an initial concentration of 0.2 × 10^8^ CFU/mL.

In the case of the co‐culture, the inocula were prepared separately. Then, 1.2 mL of *U. thermosphaericu*s inoculum was mixed simultaneously or sequentially (interval time of 12 h) with 0.8 mL of *C. taiwanensis* inoculum.

The detoxification assays were conducted by inoculating the inoculum into the synthetic or hydrolysate media for an additional 24 h detoxification treatment. These time frames represent inoculum preparation and detoxification duration, not different procedures for obtaining exudates.

### 2.2 Synthetic media composition

The synthetic media were composed of phenolic compounds (gallic acid, catechol, vanillin, and syringaldehyde), furfural, acetic acid, salts (Na_2_SO_4_, KCl, NaCl, CaCl_2_, and H_3_PO_4_), and a source of nutrients. The pH was adjusted to 7 with 4 M NaOH. Vanillin (99%), gallic acid (97.5%–102.5%), and syringaldehyde (≤98%) were supplied by Sigma‐Aldrich, while catechol (99%) was supplied from Omega Chemical Products Inc.

In the experiments using a mixture of inhibitors as a carbon source, the phenolic compounds were introduced in variable amounts into the culture media (0.3, 2.8, and 8 g/L). A stock solution containing 20 g/L of total phenolic compounds was prepared. Phenolic compounds (i.e., vanillin, catechol, syringaldehyde and gallic acid) were added at equal concentrations in the medium (i.e., 5 g/L). For preparation of media at different concentrations of phenolic compounds (i.e., 0.3, 2.8 and 8 g/L), corresponding dilutions from the stock solution were performed. The concentrations selected in this study reflect inhibitory thresholds relevant to lignocellulosic hydrolysates rather than environmental background levels. This allowed us to evaluate the functional capacity of rhizosphere microorganisms to remove toxic compounds and restore solvent production under conditions representative of industrial biomass processing.^65^

The other toxic compounds were supplemented at fixed concentrations: furfural 2 g/L, acetic acid 10 g/L and salts 0.25 M. These media were formulated to simulate the compositions of pre‐hydrolysates frequently used in industrial processes.^50^, ^51^, ^66^, ^67^, ^68^, ^69^ The phenolic compounds as well as the salts were introduced with the same concentrations. In the case of single‐compound solutions containing phenolic compounds as a sole carbon source in the absence of salts, 2, 4, and 8 g/L of the phenolic compounds were evaluated. For preparation of single‐compound solutions, stock solutions were prepared for gallic acid, vanillin, syringaldehyde and catechol separately. Then, diluted solutions at 2, 4, and 8 g/L of each phenolic compound were obtained from the stock solution.

The concentrations of toxic compounds in the medium were fixed based on the results obtained in the detoxification of a mixture of inhibitors.

Two sources of nutrients were first tested in mono‐cultures. The first medium (medium 1) contained basal salt medium (in g/L: Na_2_HPO_4_ 7, KH_2_PO_4_ 3, NaCl 0.5 and NH_4_Cl 1) and trace metal ions (in mg/L: MgSO_4_·7H_2_O 580, FeSO_4_·7H_2_O 7, CaCl_2_·2H_2_O 53.6, MnSO_4_·H_2_O 0.38, CoCl_2_·5H_2_O 0.2 and CuSO_4_·5H_2_O 0.043). The second medium (medium 2) contained (in g/L): yeast extract 5, bactopeptone 5, K_2_HPO_4_ 1 and MgSO_4_·7H_2_O 0.2. The medium exhibiting the higher performance of phenolic compounds degradation by different cultures was selected for the subsequent experiments.

### 2.3 Hemicelluloses pre‐hydrolysate preparation

The pre‐hydrolysate was obtained by steam explosion coupled with hot water pretreatment of a mixture of 60% aspen and 40% maple wood chips. The generation of the liquid pre‐hydrolysate was carried out in a pilot digester at the FPInnovations facilities (Pointe‐Claire, Québec, Canada). The details of the pre‐hydrolysis step were given in the paper of Ajao et al.^67^ The pre‐hydrolysate was then delivered to the Centre National en Électrochimie et en Technologies Environnementales (CNETE) and was characterized. It was composed of 33 g/L of total sugars, 155.1 mg/L of phenolic compounds containing 46.3 mg/L of vanillin and 108.9 mg/L of syringaldehyde, 518.3 mg/L of furfural, 82.4 mg/L of 5‐HMF and 3.63 g/L of acetic acid. The total content of phenolic compounds in the prehydrolysate was determined by the sum of the concentrations of its components. The total organic carbon content in the pre‐hydrolysate was 24.5 g/L and the total nitrogen was 0.035 g/L. The pH was adjusted from 3.5 to 7 with Ca(OH)_2_ and the resultant medium was filtered through a 1.5 μm filter prior to its use in the detoxification experiments.

### 2.4 Inhibitors removal with bacteria

The biodetoxification experiments were performed in 500 mL baffled Erlenmeyer flasks with 100 mL reaction volume composed of a carbon source (synthetic inhibitors or hemicelluloses pre‐hydrolysate) and a source of nutrients at the optimized temperature and under agitation of 200 rpm in an orbital shaker (Barnstead/Lab‐Line, MaxQ 4000 Orbital Shakers, USA). The duration of the experiments was 24 h. The detoxification experiments were performed in duplicate, and the results are the mean of two values.

### 2.5 Analytical methods

To monitor cell growth, a UV–visible spectrophotometer (Eppendorf BioPhotometer plus) was used, and the optical density was measured at 600 nm. A spectrophotometer (Pharmacia Biotech Novaspec®II, Piscatoway, NJ, USA) was used to determine the concentration of reducing sugars at 570 nm with a dinitrosalycilic acid (DNS) colorimetric method.

Phenolic compounds, furan aldehydes, and acetic acid concentrations were determined with High Performance Liquid Chromatography (HPLC) (Agilent Technologies, Germany). In the case of phenolic compounds (gallic acid, catechol, vanillin, and syringaldehyde), a diode array detector (DAD) at 313 and 280 nm and a Nucleosil C18 (150 × 4.6 mm) column were used. The solvent of separation in this case was a mixture of 15% acetonitrile and 85% phosphoric acid 10 mM, which was fed into the system at 0.8 mL/min. A 20 μL of the sample containing phenolic compounds were injected and mixed with the solvents, then passed through the column at 35°C. The retention times of gallic acid, catechol, vanillin, and syringaldehyde were 2.9, 5.5, 10.3, and 12 min, respectively. Prior to the quantification of phenolic compounds by HPLC, standard solutions of gallic acid, catechol, vanillin, and syringaldehyde were injected into the HPLC at concentrations of 0–10–50–150–200 mg/L to establish a linear calibration curve for each phenolic compound. The chromatograms of the standards (Figure A.1) and the phenolic compounds in synthetic medium (initial concentration of 8 g/L) before (Figure A.2) and after biodetoxification (Figures A.3, A.4, A.5, A.6, and A.7) are shown in the Supporting Information. For HPLC analysis, samples were prepared by dilution in the corresponding solvent to ensure that analyte concentrations fell within the calibrated quantitative range. Phenolic compounds were diluted to a maximum concentration of 500 ppm prior to analysis. Quantification was performed using external calibration curves constructed from analytical‐grade standards over the relevant concentration ranges.

Furan aldehydes (furfural and 5‐hydroxymethylfurfural) were analyzed by HPLC using a Nucleosil C18 column (150 × 4.6 mm) and a diode array detector (DAD) set at 280 nm. Elution was carried out at a flow rate of 0.8 mL·min^−1^ using a mobile phase composed of acetonitrile, acetic acid, and deionized water (84:1:15, v/v/v).

Acetic acid was analyzed using an HPLC system equipped with a diode array detector and an Inertsil ODS‐3 column (150 × 4.6 mm) maintained at 40°C. The mobile phase consisted of 1% acetonitrile in 99% KH_2_PO_4_ buffer (pH 2.8), with a flow rate of 1.25 mL·min^−1^. Quantification was based on external calibration using standard solutions.

The total nitrogen was determined by applying the Kjeldahl procedure with a Foss TecatorTM digester system and a KjeltecTM 8200 auto distillation unit.^70^

For measurement of the total organic carbon (TOC), an ASI‐L autosampler and a TOC‐L series analyzer from Shimadzu were used.^38^

### 2.6 Statistical analyses

For statistical analyses, Microsoft Excel 365 was used. Data were analyzed by one‐way analysis of variance (ANOVA), and differences among groups were considered statistically significant based on *p*‐value evaluation <0.05. All experiments were performed in triplicates, and data are presented as mean ± standard deviation.

## 3 RESULTS AND DISCUSSION

### 3.1 Detoxification procedure in synthetic media

#### 3.1.1 Effect of nutrients source on the detoxification of a mixture of inhibitors

In this part of the study, the effect of two sources of nutrients (compositions detailed in the “Material and methods” section) on the detoxification of synthetic media with pure cultures *U. thermosphaericus* and *C. taiwanensis* was evaluated. Synthetic media were composed of phenolic compounds added at different concentrations (0.3, 2.8, and 8 g/L), furfural 2 g/L, acetic acid 10 g/L and salts 0.25 M.

**FIGURE 1:**
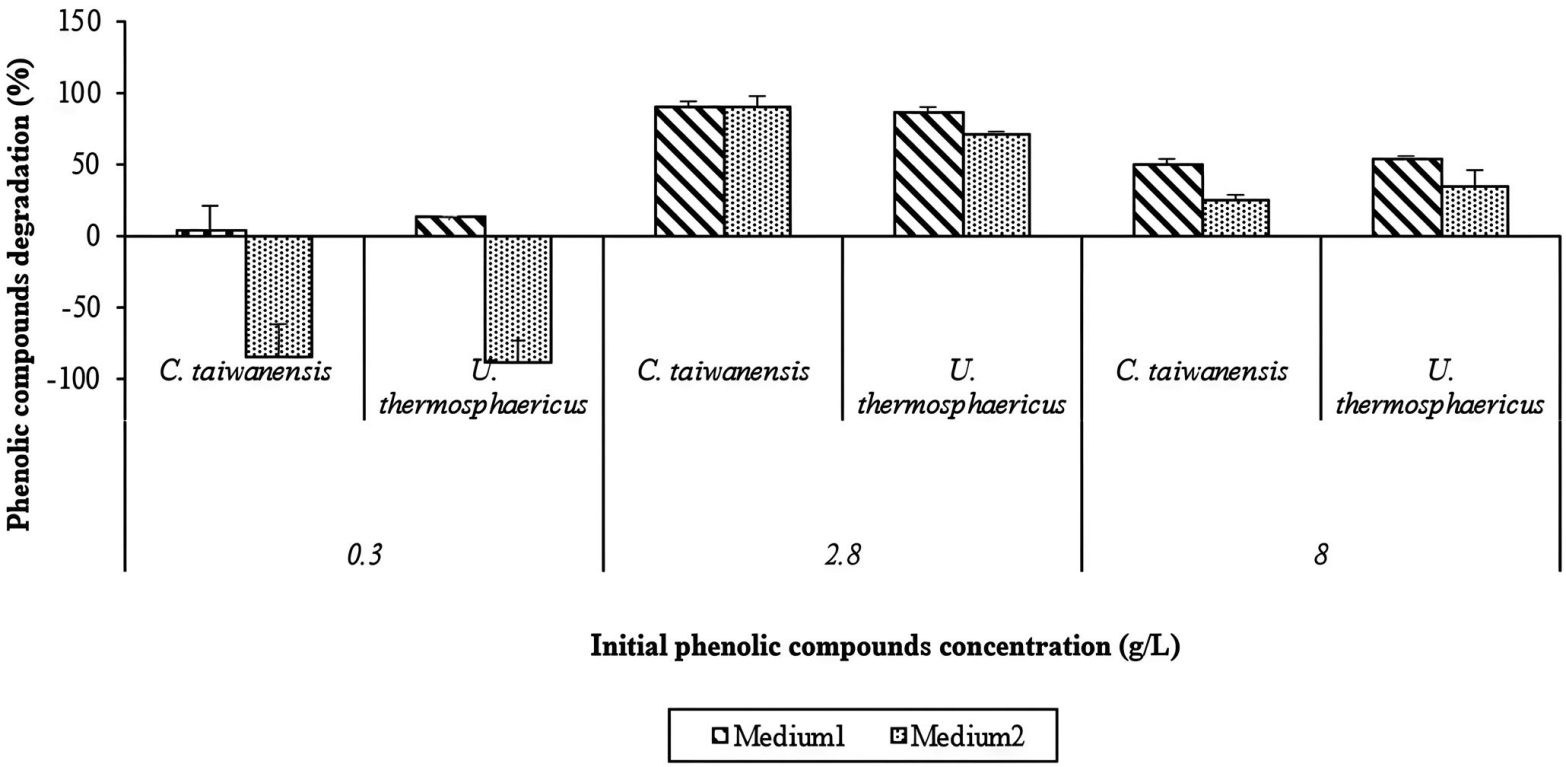
Degradation of phenolic compounds by monocultures of *U. thermosphaericus* and *C. taiwanensis* grown in two different synthetic media (basal salts with trace metals or yeast extract–bactopeptone–based medium) at 200 rpm for 24 h.

Figure 1 shows the degradation of phenolic compounds with pure cultures using the different media. At low concentration of 0.3 g/L, and using basal salt medium and trace metal ions (medium 1) as nutrient source, both cultures (*C. taiwanensis* and *U. thermosphaericus*) exhibited weak detoxification performance. The degradation of phenolic compounds did not exceed 13% with *U. thermosphaericus*. A plausible explanation of this observation is that the inhibitors were at a concentration below the threshold for expression of degrading enzymes genes. In the presence of nutrient source containing yeast extract, bactopeptone, K_2_HPO_4_ and MgSO_4_·7H_2_O (medium 2), negative values of removal were obtained, as illustrated in Figure 1. These negative values may be attributed to the accumulation of degradation intermediates in the medium.^71^ Negative removal values in medium 2 likely reflect accumulation of degradation intermediates that partially co‐elute with the parent phenolics in this complex matrix.^72^, ^73^ These intermediates can show retention times and UV spectra very similar to those of the target phenolic compounds. Ajao et al. reported that degradation of syringaldehyde could produce intermediates that co‐elute with vanillin, leading to apparent increases in the vanillin peak during treatment.^67^

Cultures performed substantially better in terms of phenolic degradation in both media when phenolic compounds were increased to 2.8 g/L. The removal efficiency was close to 90% with *C. taiwanensis* in both media (Figure 1). With the *U. thermosphaericus* culture, removal percentages were 86% and 71%, respectively, in medium 1 and medium 2. In conclusion, a significant decrease of phenolics was observed at a higher concentration of the inhibitor (2.8 g/L), compared to the results with cultures containing a lower concentration (0.3 g/L). As a result, phenolics can act as a substrate for bacteria to degrade inhibitors, which confirms the previous assumption that low concentrations of phenolics were not sufficient to trigger biodetoxification pathways. Previous studies have shown that higher phenolic concentrations can accelerate degradation by inducing redox‐mediated reactions.^71^, ^74^ Mishra et al. found that increasing syringic and gallic acid concentrations enhanced lignin degradation. They were supplemented to enhance *Coriolus Versicolor*'s detoxification of sweet sorghum bagasse.^75^ Thus, thresholds were established for the substrates to express their specific enzymes.^76^

A higher phenolic concentration of 8 g/L is also responsible for a higher extent of phenolic compounds removal. 52% of the phenolics were removed using *C. taiwanensis* using basal salt medium and trace metal ions as nutrients source (medium 1). The level of removal was similar with *U. thermosphaericus* (55% of the initial 8 g/L of phenolics). The corresponding HPLC chromatograms of the monocultures using medium 1 are presented in Figures A.6 and A.7. The initial composition is shown in Figure A.2. Using medium 2 (yeast extract, bactopeptone, K_2_HPO_4_, and MgSO_4_.7H_2_O), the bacteria degraded the phenolic compounds to a lesser extent: 24% and 34% for *C. taiwanensis* and *U. thermosphaericus*, respectively (Figure 1).

Heavy metals and trace ions present in medium 1 may act as cofactors (e.g., CuSO_4_ and MnSO_4_ ^54^) or mediators (e.g., Cu^2+^)^77^ for oxidative enzymes,^53^, ^78^ which can explain the higher degradation performance observed and justified using medium 1 for further experiments. The positive ions abundant in medium 1 can contribute to bond rupture and participate in the formation of phenoxy radicals, thus triggering degradation reactions.^66^, ^79^

After medium optimization with mono‐cultures, simultaneous and sequential cultures of *C. taiwanensis* and *U. thermosphaericus* were then tested at 41°C with medium 1 to detoxify mixed and single‐phenolic compound solutions.

#### 3.1.2 Effect of co‐culture type on the detoxification of a mixture of inhibitors

In this part of the study, simultaneous and sequential inoculation of bacteria in co‐culture was tested to detoxify synthetic media containing phenolic compounds at different concentrations (0.3, 2.8, and 8 g/L), furfural (2 g/L), acetic acid (10 g/L), and salts (0.25 M).

**FIGURE 2:**
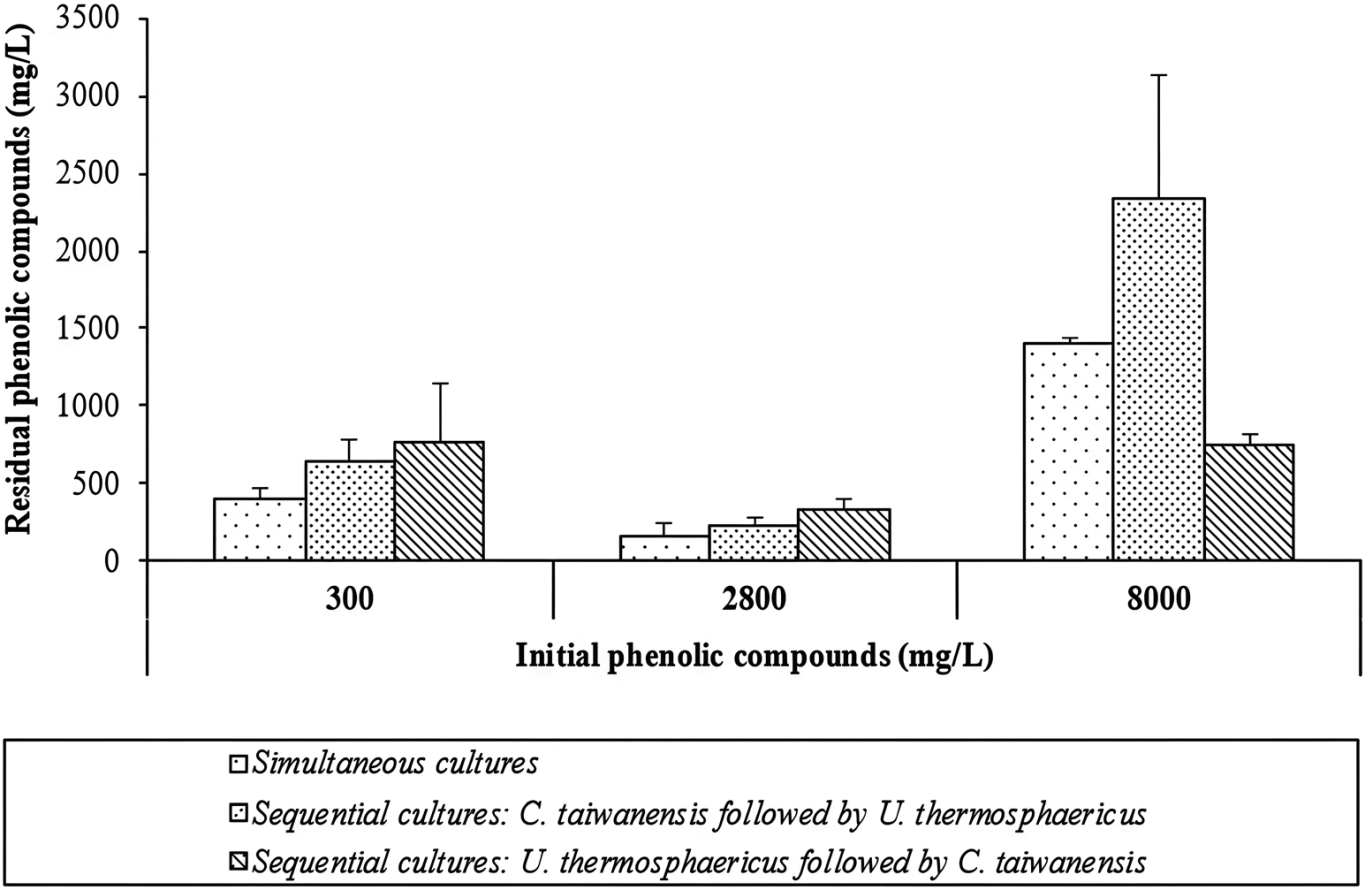
Degradation of total phenolic compounds in synthetic medium at different concentrations by simultaneous and sequential cocultures of *U. thermosphaericus* and *C. taiwanensis* at 200 rpm for 24 h.

Figure 2 shows the degradation of phenolic compounds with co‐cultures at different initial concentrations. At 0.3 g/L, the concentration increased to 0.7 g/L and 0.8 g/L for *C. taiwanensis* followed by *U. thermosphaericus* and *U. thermosphaericus* followed by *C. taiwanensis*, respectively. The concentration of phenolic compounds determined by HPLC before inoculation was about 0.5 g/L. In the simultaneous cultures, in contrast to the sequential cultures, the concentration of phenolic compounds decreased to 0.4 g/L, a decrease of 16.4%. To better understand the metabolism of the cells behind these observations, the degradation of each phenolic compound in the mixture was tested.

**FIGURE 3:**
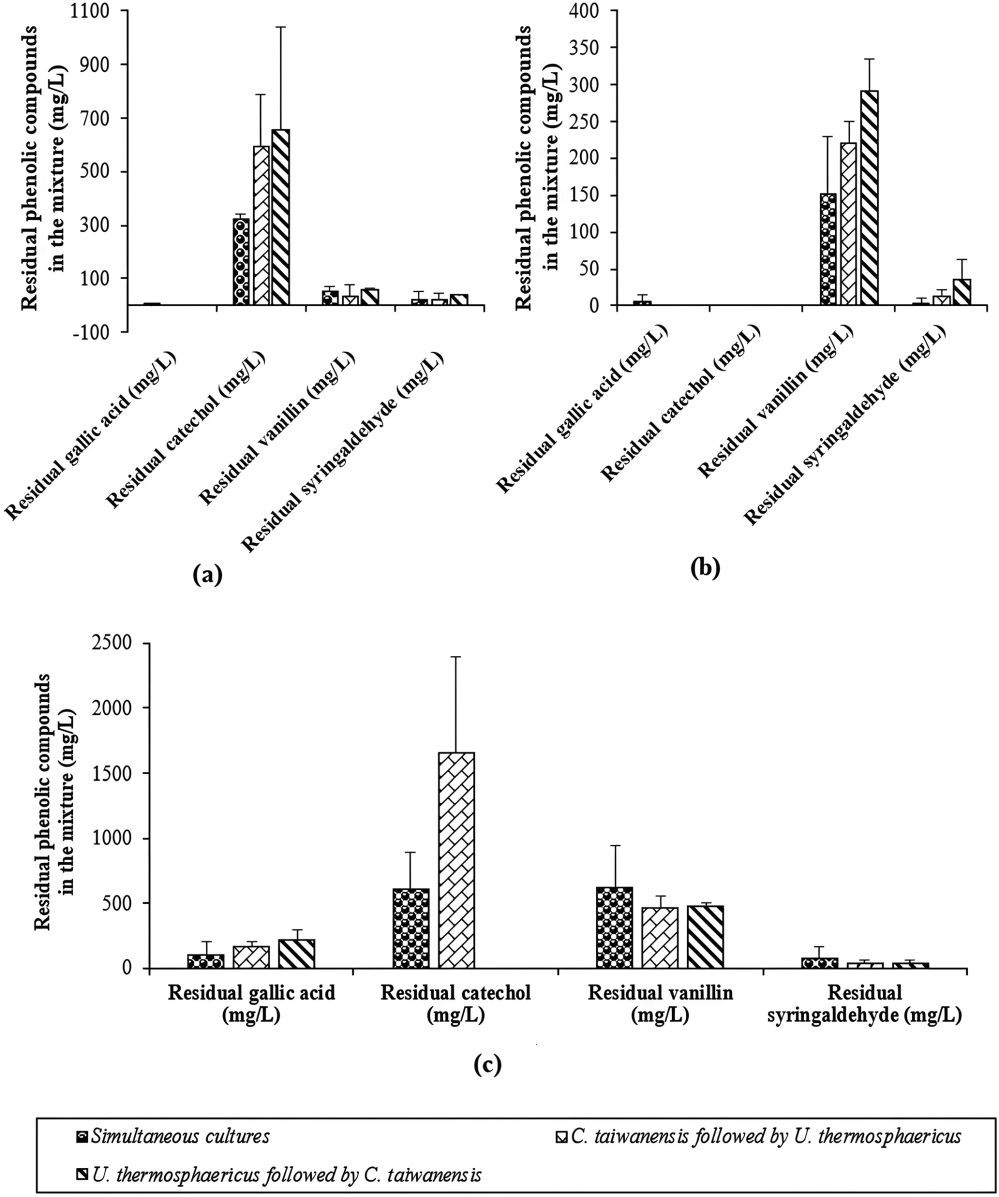
Residual individual phenolic compounds in a synthetic inhibitor mixture after 24‐h treatment with simultaneous and sequential cocultures of *U. thermosphericus* and *C. taiwanensis* at initial phenolics concentrations of (a) 0.3 g/L, (b) 2.8 g/L, and (c) 8 g/L.

At 0.3 g/L of total phenolic compounds, gallic acid was almost totally removed after 24 h of treatment with different bacteria associations (Figure 3a). Syringaldehyde was degraded to a lower extent (46%, 49%, and 7%, respectively with the simultaneous cultures, *C. taiwanensis* followed by *U. thermosphaericus* and *U. thermosphaericus* followed by *C. taiwanensis*) from an initial compound concentration in the mixture of 41.5 mg/L. In terms of vanillin degradation, its concentration decreased by 3% and 32%, respectively with the simultaneous cultures and *C. taiwanensis* followed by *U. thermosphaericus* from an initial concentration in the mixture of 52.0 mg/L. It increased to 62.0 mg/L with *U. thermosphaericus* followed by *C. taiwanensis*, corresponding to a 17% increase from the initial concentration of vanillin. Catechol concentration was slightly reduced with the simultaneous cultures (5%) and increased to 0.6 ± 0.2 g/L and 0.7 ± 0.4 g/L, respectively with *C. taiwanensis* followed by *U. thermosphaericus* and the inverse sequence (Figure 3a). Overall, at 0.3 g/L initial concentration of phenolic compounds, the simultaneous cultures showed an increased affinity to: vanillin < catechol < syringaldehyde < gallic acid, while the sequential cultures showed a higher preference to gallic acid, followed by syringaldehyde, then vanillin and to a lesser extent catechol. The removal efficiency was determined with the following equation:

$$\%\text{of phenolic compound removal}=\begin{array}{l} \text{Initial concentration}\\ -\;\text{Final concentration}/\text{Initial concentration}\\ \times 100 \end{array}$$

Gallic acid was almost totally removed with different co‐cultures, while catechol, vanillin, and syringaldehyde had a higher rate of removal with the simultaneous cultures (5%), *C. taiwanensis* followed by *U. thermosphaericus* (32%) and *C. taiwanensis* followed by *U. thermosphaericus* (49%), respectively. The increase of phenolic compound concentration in this study, mainly catechol, is similar to the results obtained in the study of Ajao et al.,^67^ where the efficiency of detoxification of phenolic compounds in single‐compound solutions or a mixture was tested with laccase enzymes. The authors stated complete removal of gallic acid followed by syringaldehyde. However, vanillin and catechol were accumulated, and negative values were reported.^67^ An explanation of the negative values for vanillin and catechol was already explained in Section 3.1.1.

A possible explanation for affinity orders is that gallic acid contains more hydroxyl groups in its structure than syringaldehyde, vanillin, and catechol, which makes it more accessible to enzymes produced by bacteria. Hence, it was observed that gallic acid was preferentially degraded by the mixed cultures followed by syringaldehyde containing two methoxy groups, one hydroxyl group, and one aldehyde group. The existence of different functional groups in gallic acid, catechol, vanillin, and syringaldehyde may contribute to different removal yields.^67^ For syringaldehyde and vanillin, there is steric hindrance by the methoxys and the aldehyde groups.^80^ Ko and Chen reported higher removal efficiencies of phenolics with methoxy and hydroxyl functional groups than phenols with methyl groups. Oxygen‐containing methoxy and hydroxyl substituents generate more stable free radicals, enhancing thus removal yields.^81^ Gallic acid contains three very reactive hydroxyl groups. This structure gives gallic acid high and fast reactivity with laccase, a key degrading enzyme.^67^ Therefore, the functional groups of the different phenolic compounds could explain the removal efficiency observed in this study.

By increasing the initial concentration of phenolic compounds to 2.8 g/L, higher degradation values up to 90%, 86%, and 81% with the simultaneous cultures, *C. taiwanensis* followed by *U. thermosphaericus* and *U. thermosphaericus* followed by *C. taiwanensis* were respectively obtained (Figure 2). The difference in individual phenolic compounds concentration in the mixture is represented in Figure 3b. Catechol and gallic acid were almost completely removed with the different co‐cultures, followed by syringaldehyde and finally by vanillin. The higher removal of syringaldehyde was achieved with the simultaneous cultures and the sequence *C. taiwanensis* followed by *U. thermosphaericus* (around 97%). In the case of vanillin, higher degradation was obtained by the simultaneous cultures (58%), then by the sequential culture, *C. taiwanensis* followed by *U. thermosphaericus* (39%), and the sequential culture *U. thermosphaericus* followed by *C. taiwanensis* (20%) (Figure 3b). The lower percentages of vanillin degradation compared to the other phenolics reported in this study could be related to the structure of vanillin. Indeed, it contains one hydroxyl group and one methoxy group, thus a small number of oxidable groups. The detoxification performance of the co‐cultures (in terms of phenolic compounds degradation) was close to the mono‐cultures (Figure 1).

Further increase of the concentration of the phenolic compounds to 8 g/L resulted in degradation up to 76%, 60%, and 87% with the simultaneous cultures, the sequential culture *C. taiwanensis* followed by *U. thermosphaericus* and the sequential culture *U. thermosphaericus* followed by *C. taiwanensis* (Figure 2), respectively. The compositional analysis of the mixture is shown in Figure 3c. The corresponding HPLC chromatograms of the different cocultures are presented in Figures A.3, A.4, A.5, and summarized in Table A.1.

Higher gallic acid removal was obtained with the simultaneous cultures and *C. taiwanensis* followed by *U. thermosphaericus* with an average of 92% ± 2.6. With the sequence of *U. thermosphaericus* followed by *C. taiwanensis*, the gallic acid concentration decreased to around 0.23 ± 0.07 g/L, which corresponded to an 87% decline from its initial value (i.e., 1.70 g/L). Regarding the degradation of catechol, total removal was obtained with the sequence *U. thermosphaericus* followed by *C. taiwanensis* after 24 h, while 63% were degraded with the simultaneous cultures. With the sequence, *C. taiwanensis* followed by *U. thermosphaericus*, a change in catechol concentration was not observed (Figure 3c) to the initial concentration (1.6 ± 0.8 g/L). The different types of consortia degraded syringaldehyde to the same extent (94% ± 2.3). Thus, at 8 g/L of phenolic compounds, the simultaneous cultures had a high affinity towards syringaldehyde and gallic acid followed by catechol and then vanillin. At the same conditions (i.e., 8 g/L), *C. taiwanensis* followed by *U. thermosphaericus* had a preference for syringaldehyde and gallic acid, followed by vanillin and finally catechol. The sequential culture *U. thermosphaericus* followed by *C. taiwanensis* showed a higher preference for catechol, followed by syringaldehyde, then gallic acid, and finally vanillin. It is noteworthy that the degradation of phenolic compounds with the different types of co‐cultures is higher than the mono‐cultures (49% with *C. taiwanensis* and 53% with *U. thermosphaericus*) at 8 g/L of initial phenolic compounds. The compositional analysis of the different cultures showed similar performances of gallic acid, syringaldehyde, and vanillin degradation with mono‐ and co‐cultures (data not shown for the mono‐cultures). Catechol removal was higher with mixed cultures (i.e., the simultaneous cultures (63%) and *U. thermosphaericus* followed by *C. taiwanensis* (100%)) than in pure cultures, which exhibited a negative percentage (data not shown). These findings are important and highlight the synergistic effects between the strains in the co‐culture when degrading a broader range of inhibitors.

In summary, similarly to the mono‐cultures, the co‐cultures showed higher performance of detoxification at 2.8 g/L initial concentration of phenolics, followed by 8 g/L and finally by 0.3 g/L. As explained above, the high concentration of phenolic compounds (8 g/L) could be toxic to cell culture. At low concentrations (0.3 g/L), bacterial competition could occur in the co‐cultures due to substrate limitation in the medium (i.e., phenolic compounds).

In the first sets of detoxification experiments with synthetic media, the removal of phenolic compounds when mixed with furfural, acetic acid, and salts was studied. However, it is important to determine the performance of mono‐ and co‐cultures to detoxify single phenolic compound media. Hence, the effect of the other inhibitors on the performance of phenolic compounds' biodegradation could be determined. Detoxification experiments of single phenolic compound solutions with pure and mixed cultures were carried out.

#### 3.1.3 Detoxification of single phenolic compound solutions

**FIGURE 4:**
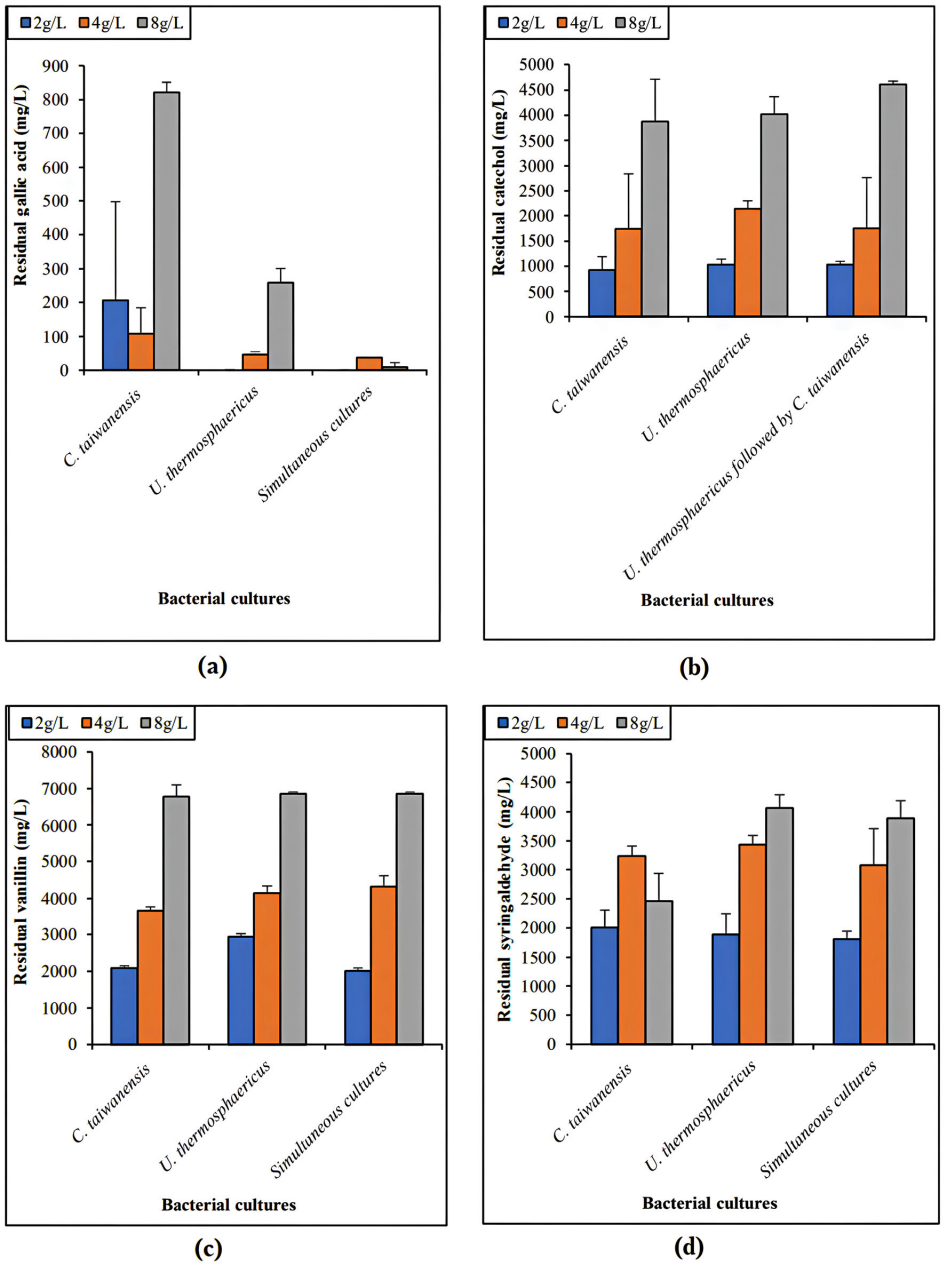
Residual phenolic compounds—(a) gallic acid; (b) catechol; (c) vanillin; (d) syringaldehyde—following 24‐h treatment of individual phenolics solutions (2, 4, and 8 g/L) with monocultures and cocultures of *U. thermosphaericus* and *C. taiwanensis*.

To investigate the performance of pure and mixed cultures for the degradation of individual phenolic compounds, single‐component solutions were prepared with different concentrations of phenolics (2, 4, and 8 g/L) and nutrient sources (medium 1). The type of co‐culture used for detoxification of each single‐component solution was selected based on the best results reported in the case of a mixture. Thus, the sequential culture of *U. thermosphaericus* followed by *C. taiwanensis* was selected for catechol solutions, while the simultaneous cultures were used for degradation of gallic acid, vanillin, and syringaldehyde. Figure 4a shows a comparison of residual gallic acid in pure and simultaneous cultures.

At 2 g/L, gallic acid was completely removed with *U. thermosphaericus* and the co‐culture, whereas *C. taiwanensis* degraded gallic acid to a final concentration of 206 mg/L. At 4 g/L, approximately 107, 46, and 37 mg/L remained in the pure cultures of *C. taiwanensis* and *U. thermosphaericus* and the co‐culture, respectively. At 8 g/L, a higher gallic acid concentration remained in the pure cultures after 24 h of treatment: 821 and 259 mg/L in *C. taiwanensis* and *U. thermosphaericus*, respectively. However, the co‐cultures had a lower final gallic acid concentration than in the pure cultures (9 mg/L). In conclusion, the simultaneous cultures showed better performance in gallic acid removal at different inhibitor concentrations, higher than values reported in mixed phenolic compounds solution (i.e., 100% vs. 90%) (Figure 3c).

Figure 4b shows the remaining catechol after 24‐hour treatment with mono‐ and co‐cultures (i.e., sequential culture *U. thermosphaericus* followed by *C. taiwanensis* with an inoculation interval of 12 h) at different initial concentrations of the phenolic compound. At 2 g/L, the pure and mixed cultures degraded catechol to a similar extent, averaging 997 mg/L. Nevertheless, catechol was completely degraded with the coculture in a mixture of phenolics at 2.8 g/L (Figure 3b). Increasing the initial concentration of catechol to 4 g/L resulted in similar degradation performance with the pure culture *C. taiwanensis* and with the coculture, with an average value of residual catechol of 1747 mg/L. At 8 g/L, the mono‐cultures showed higher degradation performance than the co‐culture, in which the residual catechol content was 4609 mg/L after 24 h of treatment, although catechol was completely removed from a mixture of phenolics with an initial concentration of 8 g/L (Figure 3c). One possible explanation for these results is the bacteria's need for salts and other carbon sources in the medium (vanillin, syringaldehyde, gallic acid, furfural, etc.) to stimulate higher production of enzymes to degrade inhibitors.

Figure 4c shows the remaining vanillin after 24 h of treatment with pure and simultaneous cultures at different initial concentrations. At 2 g/L, the residual vanillin concentration after treatment with *C. taiwanensis* and co‐culture was 2047 and 2949 mg/L with *U. thermosphaericus*. Remarkably, the final concentration of vanillin obtained with the simultaneous cultures in single‐component solutions was 13‐fold higher than that in a mixture of inhibitors (Figure 3b). These values were close to the initial concentration of vanillin, illustrating the inability of these strains to counteract the inhibitory effects of the single phenolic compounds solutions. At 4 g/L of vanillin, lower levels remained in the monoculture *C. taiwanensis* (3656 mg/L) compared with the culture of *U. thermosphaericus* (4144 mg/L) and the co‐culture (4320 mg/L). At 8 g/L, the different cultures removed vanillin almost equally up to a final concentration of about 6824 mg/L after 24 h. It is important to highlight that the final concentration of vanillin contained in a mixture of phenolic compounds with an initial concentration of 8 g/L was about 617 mg/L after 24 h with the co‐culture (Figure 3c), which is 11 times less than that obtained in a single component solution under the same operating conditions. These results confirm that the bacteria require other carbon sources (furfural, other phenolic compounds, etc.) in the medium to stimulate the production of enzymes that degrade inhibitors.

Figure 4d shows the residual amount of syringaldehyde after treatment with pure and simultaneous cultures at different concentrations. At 2 g/L of syringaldehyde, the final concentrations were 2009, 1886, and 1810 mg/L, respectively with *C. taiwanensis*, *U. thermosphaericus*, and the co‐culture. These values were close to the initial concentration of syringaldehyde in the medium (2 g/L), demonstrating a low degradation performance at this inhibitory level. Only 4.27 mg/L of syringaldehyde remained from 2.8 g/L mixture of phenolics with the simultaneous cultures after 24 h. Previous studies on the strains *C. taiwanensis* and *U. thermosphaericus* demonstrated their potential to produce catalase, laccase, peroxidase, and oxidase enzymes.^28^, ^52^ These enzymes participate in the oxidation of the inhibitors. At 4 g/L of syringaldehyde, 3239, 3431, and 3079 mg/L remained after treatment with respectively *C. taiwanensis*, *U. thermosphaericus*, and the simultaneous cultures. These residual concentrations are close to the initial values, indicating a low performance of detoxification with the different cultures in the presence of 4 g/L of syringaldehyde. At 8 g/L, a reduction of syringaldehyde concentration to 2466 mg/L with *C. taiwanensis*, 4062 mg/L with *U. thermosphaericus*, and 3892 mg/L with the simultaneous cultures was observed. Compared to the results at lower initial concentrations of syringaldehyde (2 and 4 g/L), the degradation performances with the different cultures at 8 g/L were higher (Figure 4d). This observation was in agreement with the results obtained in a mixture of inhibitors. Phenolic compounds were potential substrates for the bacteria requiring increased concentrations for their metabolism. Furthermore, it was observed that at 8 g/L of syringaldehyde, *C. taiwanensis* exhibited a higher performance of degradation than *U. thermosphaericus* and the co‐culture. This could be explained by the high affinity of the strain and its enzymes to syringaldehyde at high concentrations (i.e., 8 g/L). Nevertheless, the biodegradation performance in single‐component solutions is lower than that in a mixture containing 8 g/L of total phenolic compounds, where 94% of syringaldehyde was eliminated.

In summary, bacterial cultures preferentially degraded gallic acid in single‐component solutions, mainly at low concentrations (i.e., 2 and 4 g/L). Catechol represents the second most degraded phenolic compound. Weak performances of biodetoxification were observed with vanillin degradation at different concentrations and with syringaldehyde at 2 and 4 g/L. These concentrations were inhibitory for the bacteria in the absence of stimuli that act synergistically to promote the production of degradative enzymes^67^ or that react directly (as in the case of salts) with phenols to generate radicals and initiate oxidation reactions.^69^ The higher affinity for gallic acid, followed by catechol, then syringaldehyde and vanillin, could be related to the number of alcohol groups. Reduced molecules are oxidized to free radicals that contribute to further polymerization or degradation.^70^ These observations prompted the evaluation of the mono‐ and co‐cultures in the detoxification of hemicellulose prehydrolysate containing a mixture of inhibitors.

### 3.2 Optimization of hemicelluloses pre‐hydrolysate detoxification

**FIGURE 5:**
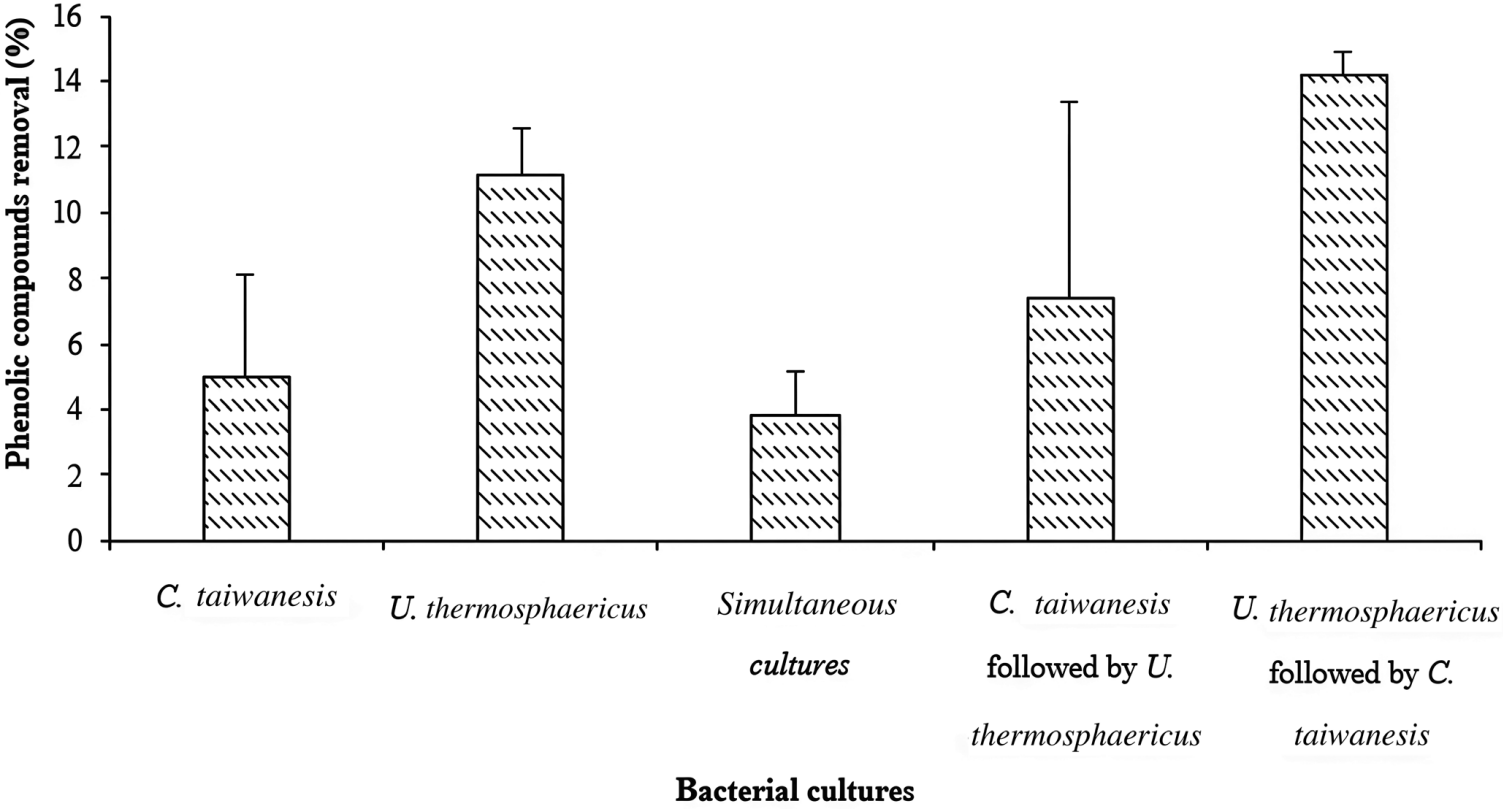
Percentage removal of total phenolic compounds from undiluted hemicellulose prehydrolysate after 24‐h of treatment at 41°C and 200 rpm.

In this part of the study, the performance of the different cultures tested for detoxification of synthetic media was evaluated in the case of undiluted hemicellulose pre‐hydrolysate. The prehydrolysate was obtained by steam explosion combined with hot water pretreatment. Pure cultures (*C. taiwanensis* and *U. thermosphaericus*) and their co‐cultures (simultaneous cultures and sequential cultures with an inoculation interval of 12 h) were used for biodetoxification. The concentration of phenolic compounds (gallic acid, catechol, vanillin, and syringaldehyde) was determined by HPLC before and after the 24‐h treatment, and the percent removal at 41°C and 200 rpm was calculated. The results are shown in Figure 5.

Using the *C. taiwanensis* monoculture, a 5% reduction in phenolic compounds was achieved after 24 h. This percentage was doubled by applying the monoculture *U. thermosphaericus*. Simultaneous inoculation of both strains contributed to a 3.8% reduction. In contrast, when the bacteria were inoculated sequentially, phenolics degradation increased to 7.4% and 14% for the sequence *C. taiwanensis* followed by *U. thermosphaericus* and for the sequence *U. thermosphaericus* followed by *C. taiwanensis*, respectively. *U. thermosphaericus* showed higher performance in degrading phenolic compounds in the prehydrolysate than *C. taiwanensis*. To the best of our knowledge, in this study, we studied for the first time the ability of the strain *U. thermosphaericus* to degrade monomeric phenolic compounds. Okuda et al.^37^ used this bacterium for detoxification of wood hydrolysate and reported degradation of furfural and HMF only. The poor degradability of *C. taiwanensis* might be related to an intolerance of this strain to the concentration of phenolic compounds in the prehydrolysate. Indeed, *C. taiwanensis* was evaluated for phenol degradation, and a maximum of 900 mg/L,^39^ 2200 mg/L^44^ and 3200 mg/L^43^ were degraded with a glycerol‐supplemented batch culture, a fed‐batch culture, and continuous culture, respectively. The initial concentration of phenolic compounds in the prehydrolysate used in our study was 155 mg/L. A possible explanation for this result is the simultaneous presence of other inhibitors in the prehydrolysate at high concentrations, including furfural, acetic acid, salts, 5‐HMF, and so forth, and there might be synergistic inhibition. The lowest removal results were obtained when both strains were inoculated simultaneously, although *U. thermosphaericus* inoculated individually gave better removal. This result could be due to the dominance of *C. taiwanensis*, which is characterized by its low detoxification ability of the prehydrolysate, thus preventing the contribution of *U. thermosphaericus*. Moreover, bacterial death of *C. taiwanensis* was expected in such mixed cultures due to the extremely inhibitory environment. Dead cells limited the ability of *U. thermosphaericus* to access its substrates. The best results obtained with the sequence U. thermosphaericus followed by *C. taiwanensis* could be due first to the access of *U. thermosphaericus* and its enzyme system to the nutrients and phenolic compounds and then to the degradation of the toxic compounds to less toxic products or a concentration not inhibitory to *C. taiwanensis*.

**FIGURE 6:**
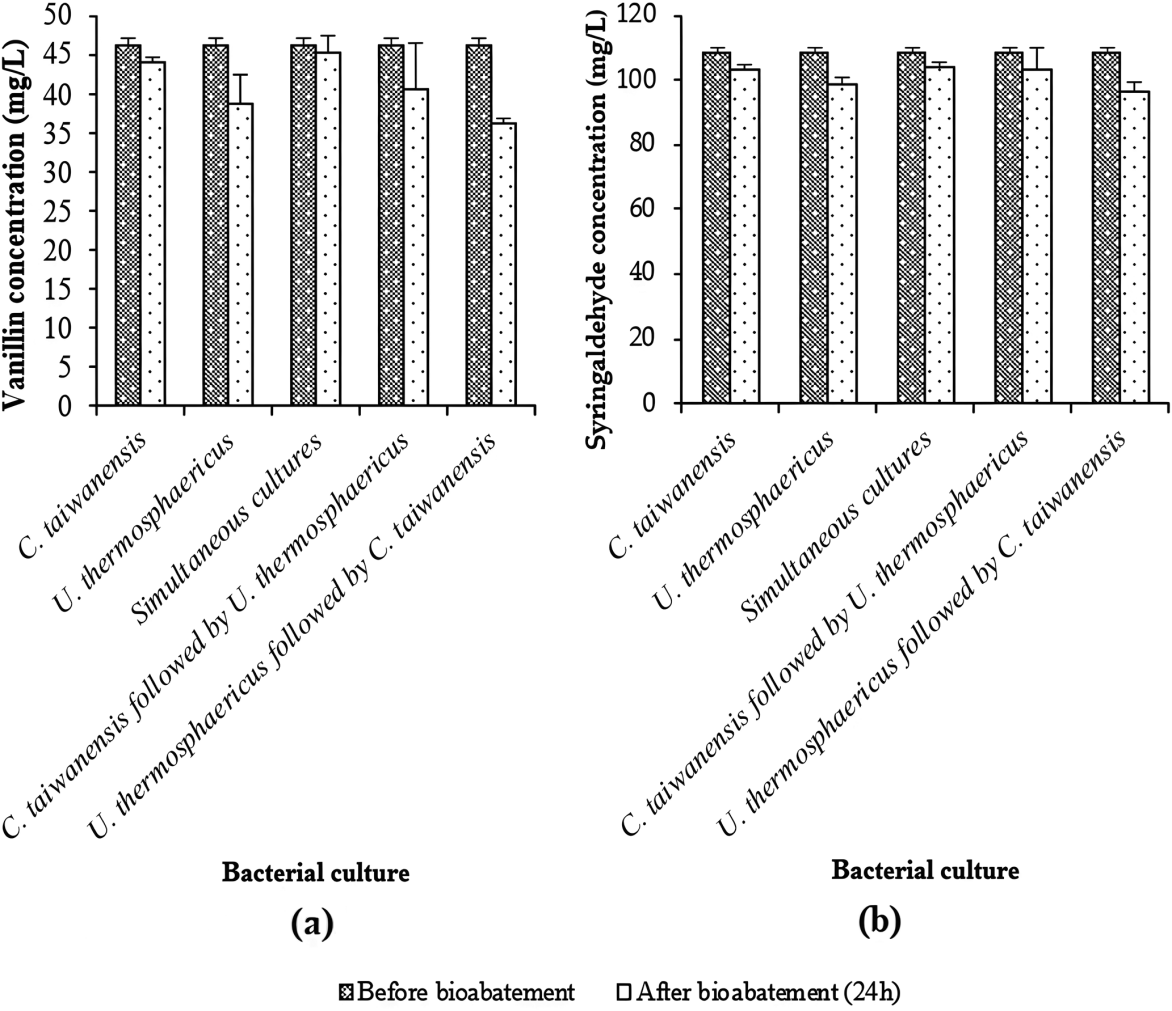
Residual concentrations of individual phenolic compounds, (a) Vanillin and (b) Syringaldehyde, present in undiluted hemicellulose prehydrolysate following 24‐h treatment with monocultures and simultaneous and sequential cocultures of *U. thermosphaericus* and *C. taiwanensis*.

To better understand the metabolism of phenolic compounds, their concentrations in the treated prehydrolysate were characterized. The results shown in Figure 6 indicate that the monoculture of *C. taiwanensis* removed 4.65% and 5.19% of vanillin and syringaldehyde, respectively. *U. thermosphaericus* removed 16.2% of vanillin and 9% of syringaldehyde, while the simultaneous cultures eliminated 1.85% of vanillin and 4.67% of syringaldehyde. Sequential culture of *C. taiwanensis* followed by *U. thermosphaericus* resulted in 12.3% and 5.22% of vanillin and syringaldehyde elimination, respectively. Better results were obtained with the sequential culture of *U. thermosphaericus* followed by *C. taiwanensis*, where vanillin and syringaldehyde removals were 27% and 11%, respectively. Based on these results, the monoculture of *U. thermosphaericus* and the sequential culture of *U. thermosphaericus* followed by *C. taiwanensis* showed higher removal of phenolic compounds.

It is notwithstanding to mention that no peroxidase enzymes were added in our system, and the experiments were conducted under controlled biological conditions, making the contribution of mineral oxidants such as MnO_2_ or Fe oxides unlikely. Moreover, we previously tested iron‐based flocculants as an alternative detoxification method, but the highest Acetone Butanol Ethanol (ABE) yields were consistently obtained with the same biodetoxified hydrolysate,^65^ confirming that the observed detoxification effect originates from biological rather than abiotic oxidation.

## 4 CONCLUSION

In this work, an optimized co‐culture of *C. taiwanensis* and *U. thermosphaericus* was developed and tested for its efficiency in degrading the major phenolic compounds (gallic acid, catechol, vanillin, and syringaldehyde) in hardwood prehydrolysate. At an initial phenolic concentration of 2.8 g/L, the new co‐culture showed excellent degradation efficiency up to 90% in synthetic media. The different co‐cultures have a higher affinity for gallic acid and catechol, followed by syringaldehyde and vanillin. Pure, simultaneous, and sequential cultures were used to detoxify real prehydrolysate. For phenolic compounds degradation, the best results were obtained with the sequential culture *U. thermosphaericus*, followed by *C. taiwanensis*, although only 14% degradation was achieved. Therefore, additional parameters should be evaluated to optimize the co‐culture and improve its performance in detoxifying lignocellulosic pre‐hydrolysates.

## AUTHOR CONTRIBUTIONS

**Mariem Theiri:** Writing – original draft; writing – review and editing; methodology. **Mariya Marinova:** Supervision, review and editing. **Hassan Chadjaa:** Supervision, review and editing. **Mario Jolicoeur:** Supervision, review and editing.

## FUNDING INFORMATION

This work was supported by a grant from the College‐University I2I Program of the Natural Sciences and Engineering Research Council of Canada (NSERC) (grant number 437803‐12) of Dr. M. Marinova, the NSERC Industrial Research Chair for Colleges in Bioprocess and Fermentation Technology of Dr. H. Chadjaa, and the NSERC Discovery Grant (# 380070‐08) of Dr. M. Jolicoeur.

## CONFLICT OF INTEREST STATEMENT

The authors declare no conflicts of interest. The funding bodies and material suppliers had no contribution to the experimental design in this work, data collection, analysis, or interpretation; manuscript preparation; or the decision to publish the results.

## Supporting information


Data S1:

## Data Availability

The data supporting the findings of this study are available from the corresponding author upon reasonable request.
