# Supplementary material for: Development of a co‐culture of Ureibacillus thermosphaericus and Cupriavidus taiwanensis for inhibitors removal from hemicellulose prehydrolysate
Source: Biotechnol Prog. 2026 Jan 19;42(2):e70107. doi: 10.1002/btpr.70107 (PMC13055127; doi:10.1002/btpr.70107)
Supplement: Supplementary file 1 — Data S1: [file BTPR-42-e70107-s001.docx]

**Supplementary Materials. Development of a co-culture of *Ureibacillus thermosphaericus* and *Cupriavidus taiwanensis* for inhibitors removal from hemicellulose pre-hydrolysate**

Mariem Theiri^1,2^, Mariya Marinova^3^, Hassan Chadjaa^2^, Mario Jolicoeur^1^

^1^ Research Laboratory in Applied Metabolic Engineering, Department of Chemical Engineering, Polytechnique Montréal, J.-A.-Bombardier Pavilion, 2900 Édouard-Montpetit Blvd., Montréal, QC, H3T 1J4, Canada

^2^ Centre National en Électrochimie et en Technologies Environnementales, 2263 avenue du Collège, Shawinigan, QC, G9N 6V8, Canada

^3^ Department of Chemistry and Chemical Engineering, Royal Military College of Canada, 13 General Crerar Crescent, Kingston, ON, K7K 7B4, Canada


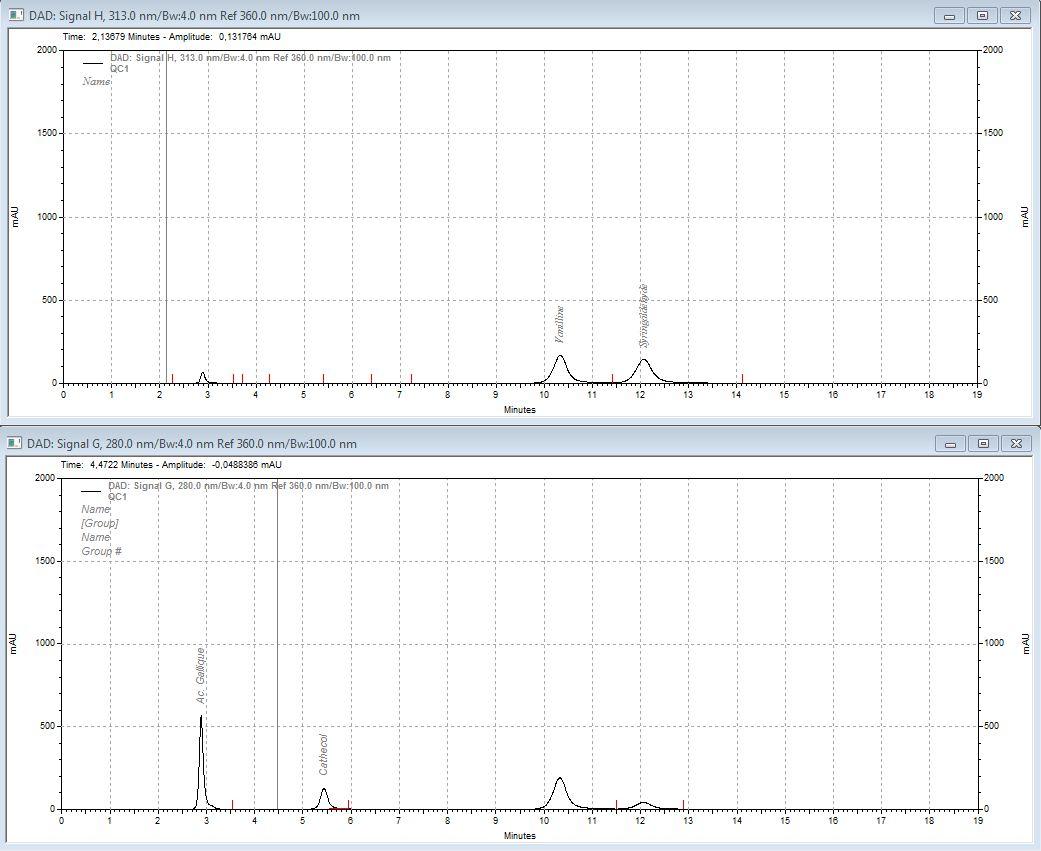


**Figure A. 1.** Chromatogram of the standards of gallic acid, catechol, vanillin and syringaldehyde.


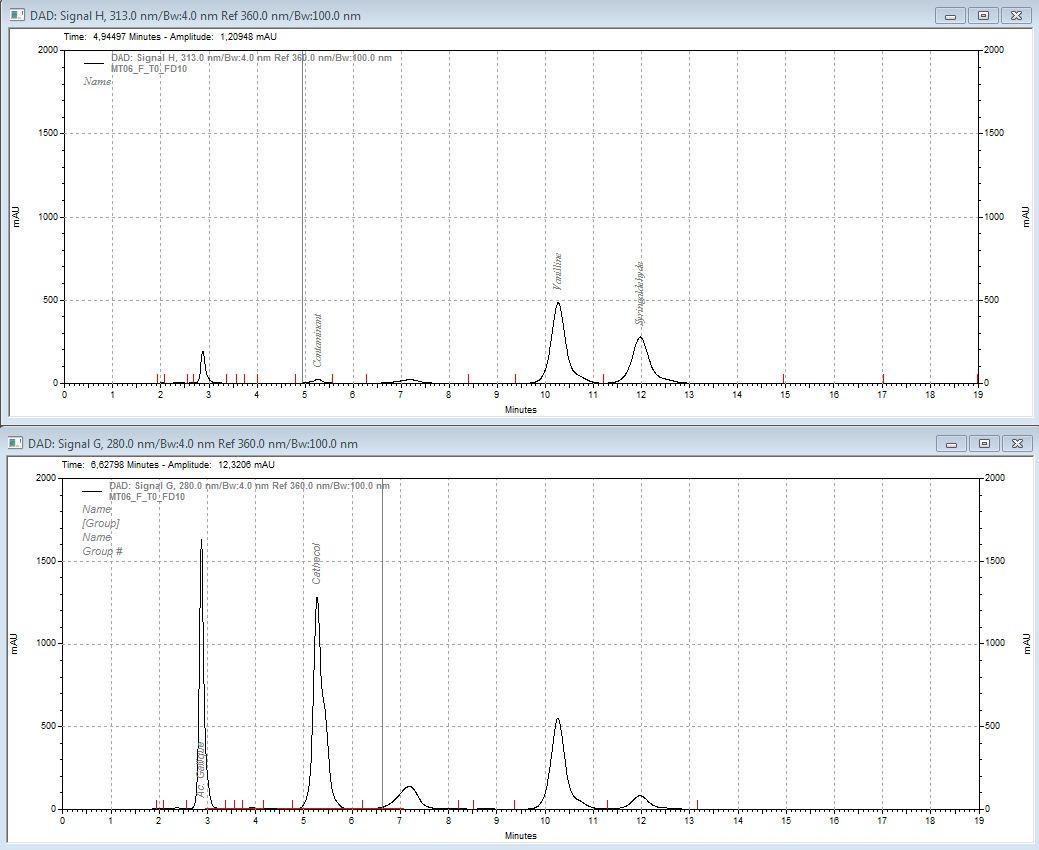


**Figure A. 2.** Chromatogram of phenolic compounds in the initial composition of synthetic medium composed of: 2 g/L furfural, 10 g/L acetic acid, 0.25 M salts and 8 g/L phenolic compounds.


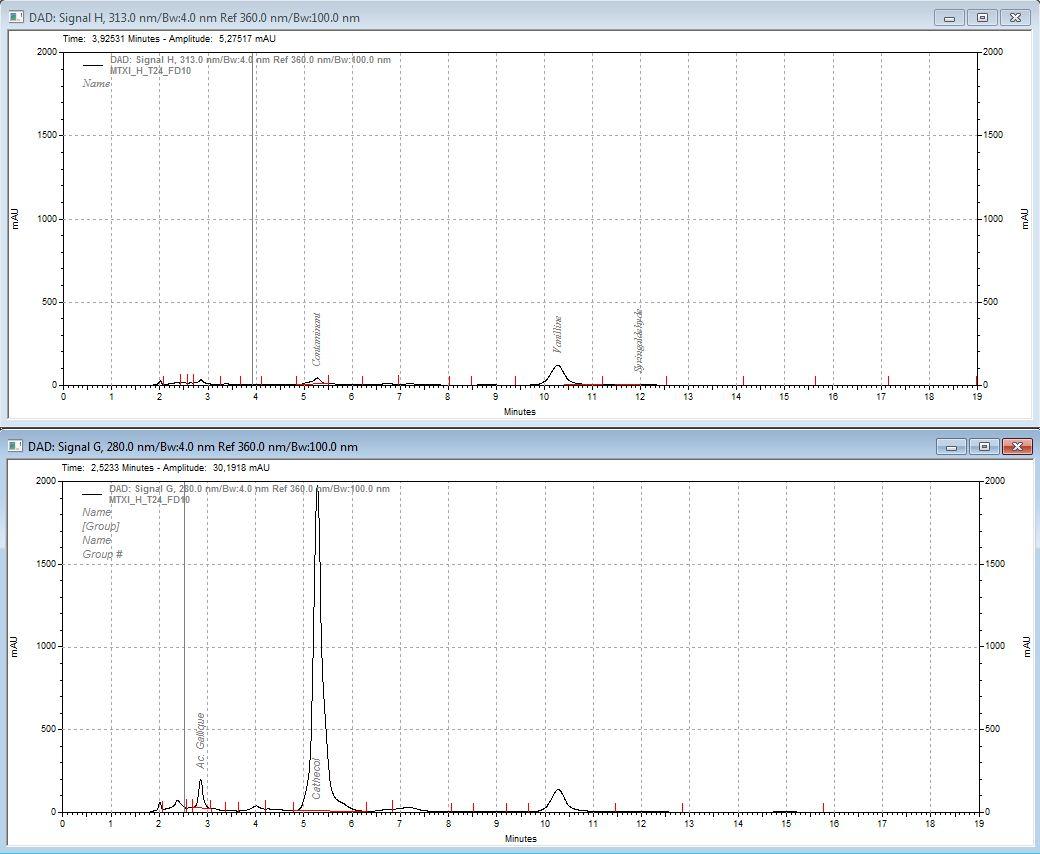


**Figure A. 3.** Chromatogram of phenolic compounds in the detoxified synthetic medium (after 24 h) with simultaneous cultures. The initial composition of synthetic medium was: 2 g/L furfural, 10 g/L acetic acid, 0.25 M salts and 8 g/L phenolic compounds.


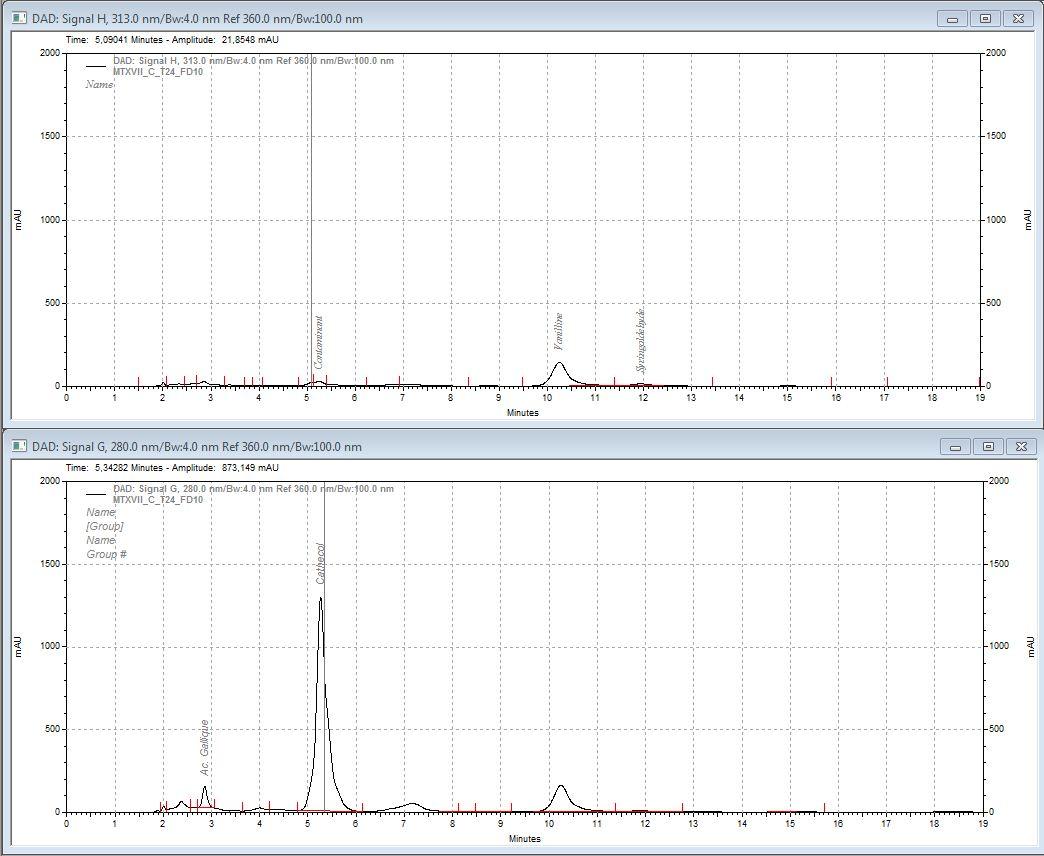


**Figure A. 4.** Chromatogram of phenolic compounds in the detoxified synthetic medium (after 24 h) with sequential cultures *C. taiwanensis* followed by *U. thermosphaericus*. The initial composition of synthetic medium was: 2 g/L furfural, 10 g/L acetic acid, 0.25 M salts and 8 g/L phenolic compounds.

*
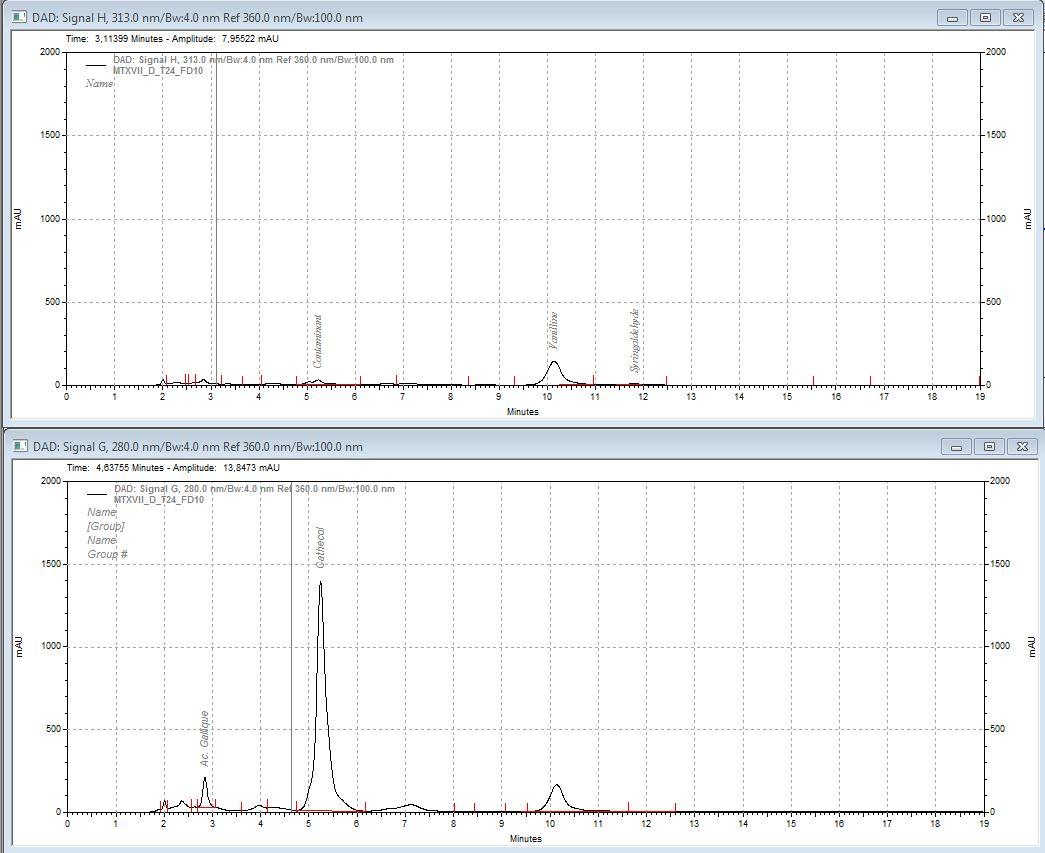
*

**Figure A. 5.** Chromatogram of phenolic compounds in the detoxified synthetic medium (after 24 h) with sequential cultures *U. thermosphaericus* followed by *C. taiwanensis*. The initial composition of synthetic medium was: 2 g/L furfural, 10 g/L acetic acid, 0.25 M salts and 8 g/L phenolic compounds.


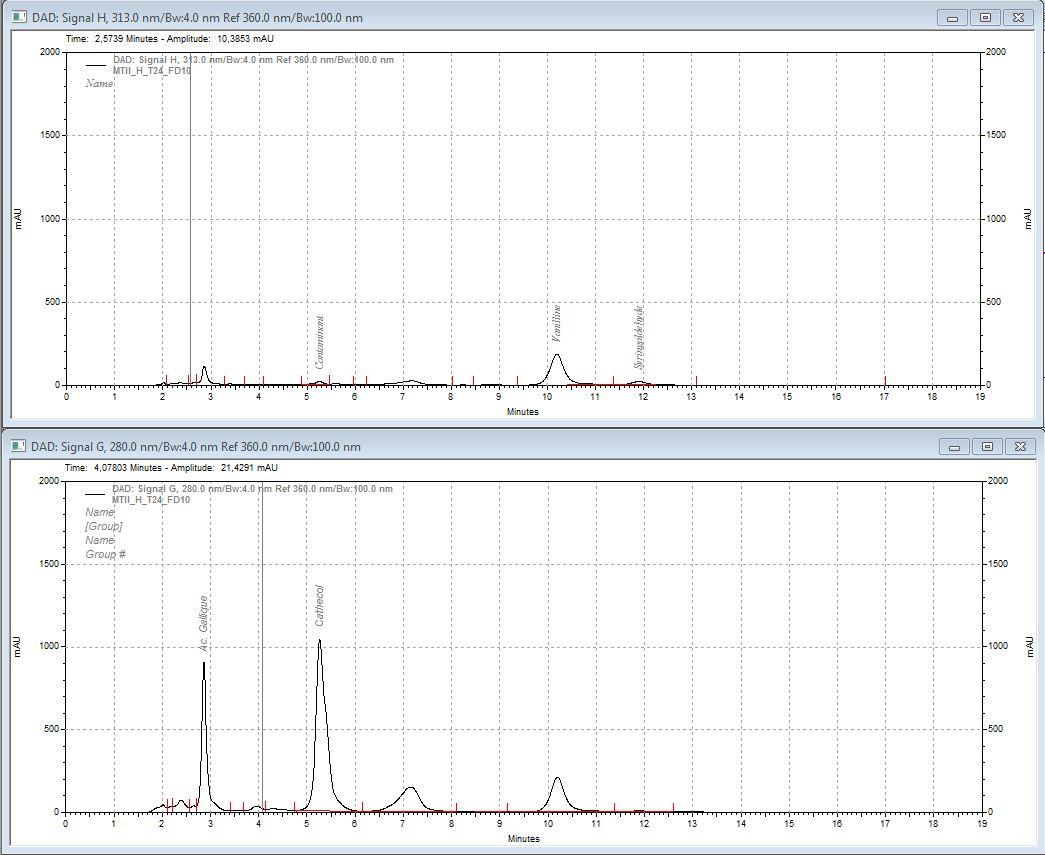


**Figure A. 6.** Chromatogram of phenolic compounds in the detoxified synthetic medium (after 24 h) with the monoculture *C. taiwanensis*. The initial composition of synthetic medium was: 2 g/L furfural, 10 g/L acetic acid, 0.25 M salts and 8 g/L phenolic compounds.


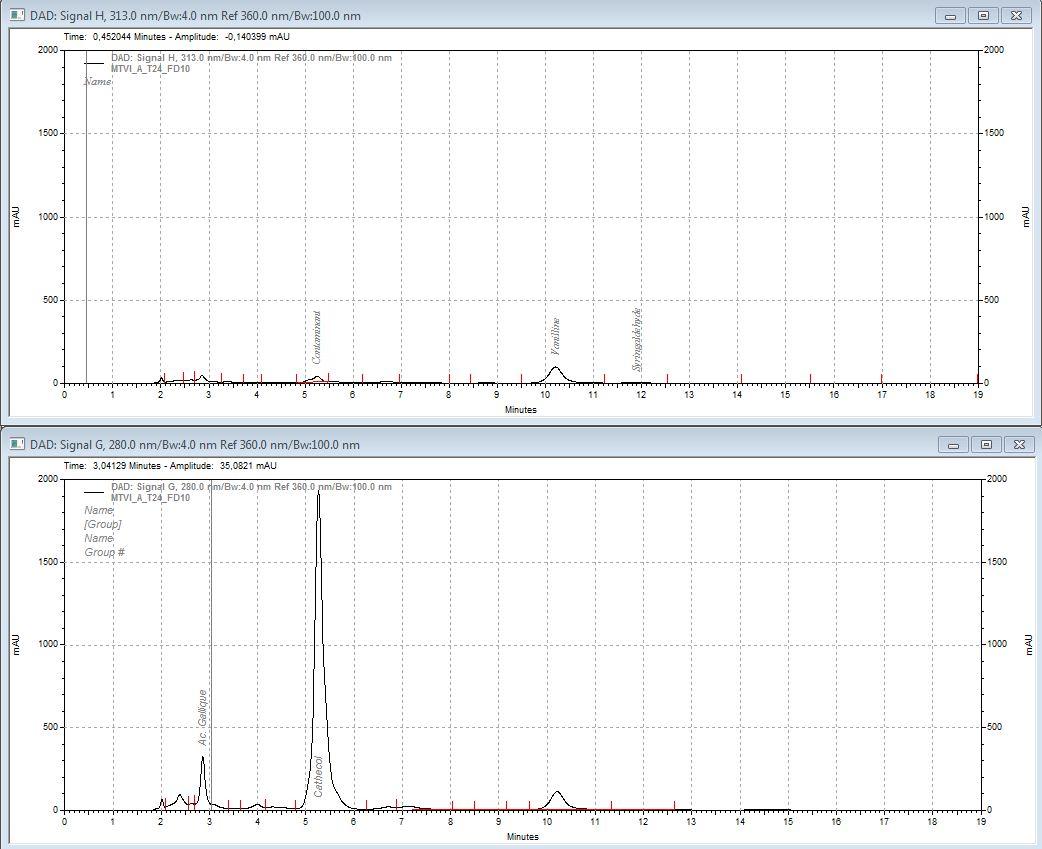


**Figure A. 7.** Chromatogram of phenolic compounds in the detoxified synthetic medium (after 24 h) with the monoculture *U. thermosphaericus*. The initial composition of synthetic medium was: 2 g/L furfural, 10 g/L acetic acid, 0.25 M salts and 8 g/L phenolic compounds.

**Table A. 1.** Composition of synthetic media before and after 24 h of detoxification with the different cultures.

| **Molarities (10^-3^ mol/L)** | | **Vanillin** | **Syringaldehyde** | **Gallic acid** | **Catechol** |
| --- | --- | --- | --- | --- | --- |
| **Initial composition** | | 10.45 | 5.72 | 10.75 | 17.13 |
| **Final composition with different cultures** | **Simultaneous cultures** | 2.56 | 0.10 | 1.05 | 16.24 |
|  | ***C. taiwanensis* followed by *U. thermosphaericus*** | 3.48 | 0.34 | 0.77 | 19.79 |
|  | ***U. thermosphaericus* followed by *C. taiwanensis*** | 3.27 | 0.15 | 1.05 | 0.00 |
|  | ***C. taiwanensis*** | 4.02 | 0.47 | 6.57 | 25.13 |
|  | ***U. thermosphaericus*** | 2.09 | 0.09 | 2.80 | 16.74 |
